# Publisher Correction: Endogenous formaldehyde is a memory-related molecule in mice and humans

**DOI:** 10.1038/s42003-019-0738-2

**Published:** 2019-12-19

**Authors:** Li Ai, Tao Tan, Yonghe Tang, Jun Yang, Dehua Cui, Rui Wang, Aibo Wang, Xuechao Fei, Yalan Di, Xiaoming Wang, Yan Yu, Shengjie Zhao, Weishan Wang, Shangying Bai, Xu Yang, Rongqiao He, Weiying Lin, Hongbin Han, Xiang Cai, Zhiqian Tong

**Affiliations:** 1 Alzheimer’s Disease Center, Beijing Institute for Brain Disorders; Center for Brain Disorders Research, Capital Medical University, Beijing, 100069 China; 2 Department of Physiology and Biophysics School of Medicine & Biomedical Sciences, State University of New York at Buffalo, Buffalo, NY 14214 USA; 3 Sichuan Provincial Hospital for Women and Children, Chengdu, 610000 China; 4 Institute of Fluorescent Probes for Biological Imaging, School of Chemistry and Chemical Engineering, School of Materials Science and Engineering, University of Jinan, Shandong, 250022 China; 5 Department of Radiology, Peking University Third Hospital, Beijing, China; 6 Key Laboratory of Magnetic Resonance Imaging Equipment and Technique, Beijing, 100191 China; 7 Beijing Boai Hospital, China Rehabilitation Research Center, Beijing, 100068 China; 8 Department of Physiology, Southern Illinois University School of Medicine, Carbondale, IL 62901 USA; 9 Section of Environmental Biomedicine, Hubei Key Laboratory of Genetic Regulation and Integrative Biology, College of Life Sciences, Central China Normal University, Wuhan, 430079 China; 10 State Key Laboratory of Brain & Cognitive Science, Institute of Biophysics, CAS Key Laboratory of Mental Health, University of Chinese Academy of Sciences (UCAS), Beijing, 100101 China

**Keywords:** Hippocampus, Alzheimer's disease, Energy metabolism

Correction to: *Communications Biology* 10.1038/s42003-019-0694-x, published online 29 November 2019.

In the original published version of the manuscript, an error was introduced in the abstract. The abstract incorrectly stated that “high formaldehyde concentrations gradually inactivate the NMDA receptor by cross-linking NR1 subunits to NR2B via the C232 residue”. Instead, the abstract should indicate that elevated formaldehyde enhances NMDA currents “via the C232 residue of the NMDA receptor”. The error has been corrected in the HTML and PDF versions of the article.

