# Supplementary material for: Endogenous formaldehyde is a memory-related molecule in mice and humans
Source: Commun Biol. 2019 Nov 29;2:446. doi: 10.1038/s42003-019-0694-x (PMC6884489; doi:10.1038/s42003-019-0694-x)
Supplement: Supplementary file 1 — Supplementary Information [file 42003_2019_694_MOESM1_ESM.pdf]

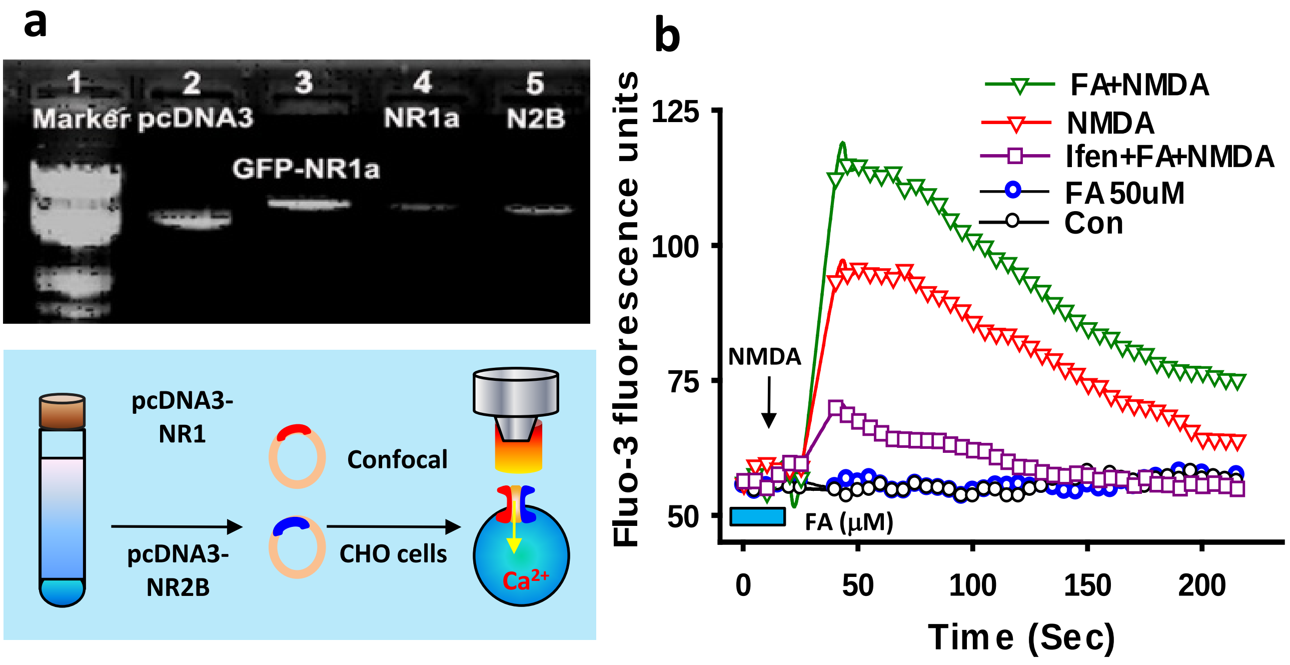

Supplementary Figure 1. Formaldehyde (FA) at 0.05 mM enhances NMDA-induced intracellular Ca^{2+} influx in the cultured GFP-NR1/NR2B transfected CHO cells. (**a**) The scheme for transfecting the plasmids of GFP-NR1/NR2B into CHO cells. (**b**) Intracellular Ca^{2+} influx measured using the probe Fluo-3 under laser scanning confocal microscopy. Ifen: Ifenprodil (an specifical antagonist of NR2B).

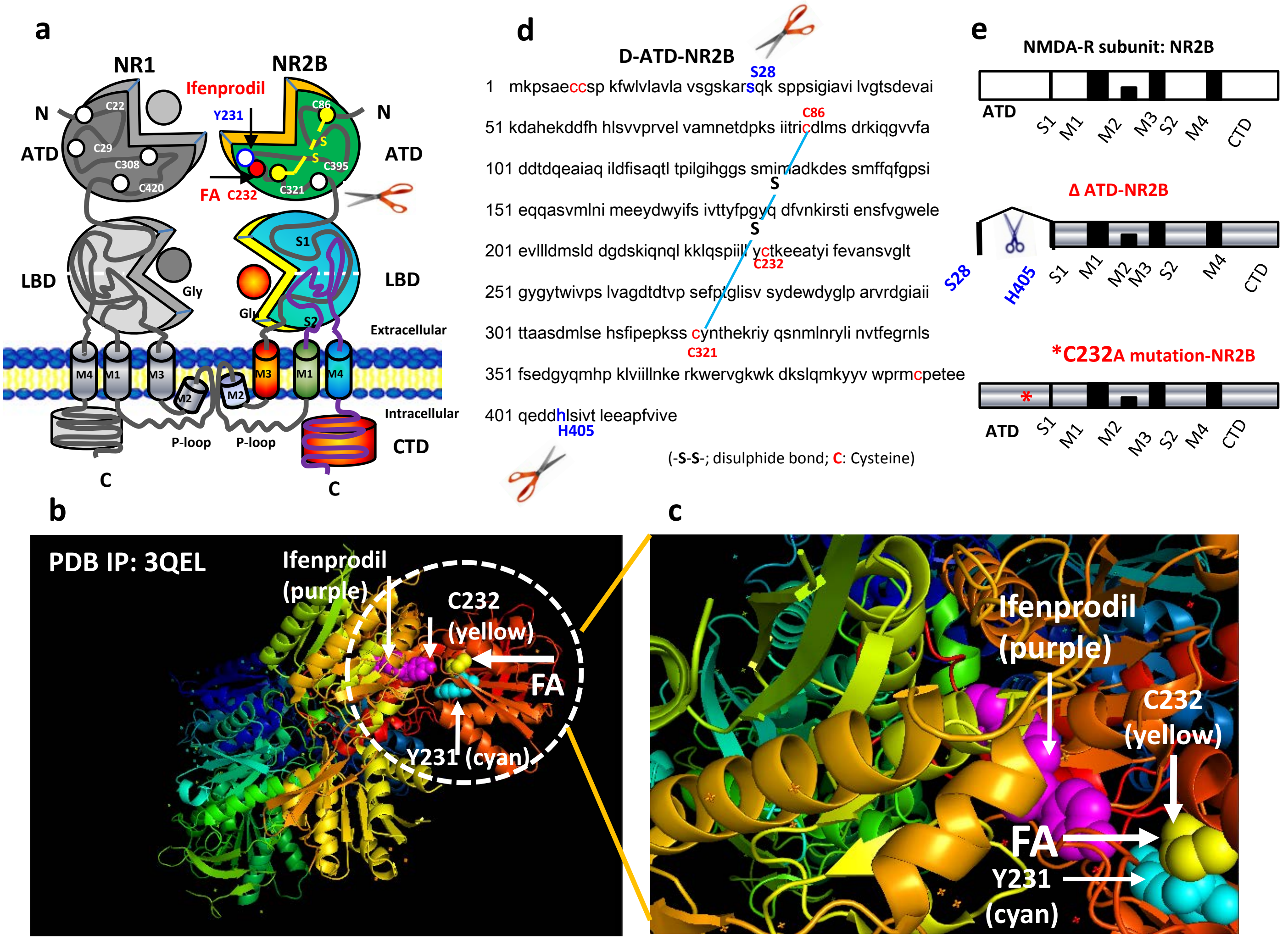

Supplementary Figure 2. A specifical NR2B antagonist- Ifenprodil binds with Y231 and C232 residues to prevent the active FA connection with C232 site of N2B. (a) The model of the complex of NR1 and NR2B. (**b**) The 3D crystal structure of Ifenprodil-binding with Y231 and C232 residues of NR1/NR2B (PDB IP: 3QEL). (**c**) The model of FA-binding with C232 of NR2B. (**d**) The sequences of deleting amino-terminal domain (D-ATD) of rat NR2B. (**e**) The model of deleting ATD of NR2B.

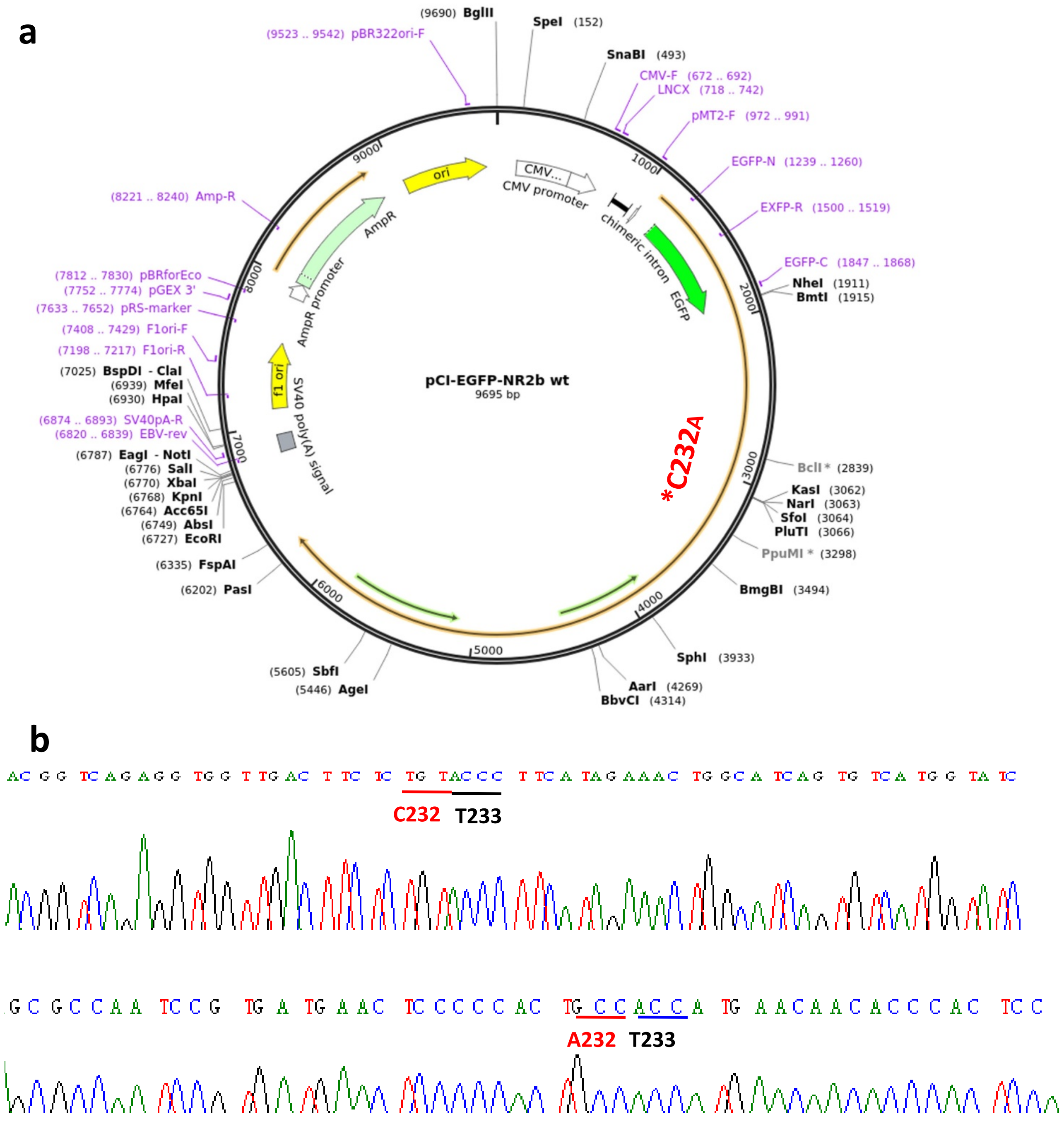

Supplementary Figure 3. The identification of mutation in C232A site of NR2B of wild-type rat. (a) The plasmid of pci-EGFP-NR2B (9695 bp). (**b)** The DNA sequence of mutation in C232A (Cys codon: TGT; Ala codon: GCC) site of NR2B examined by gene sequencing.

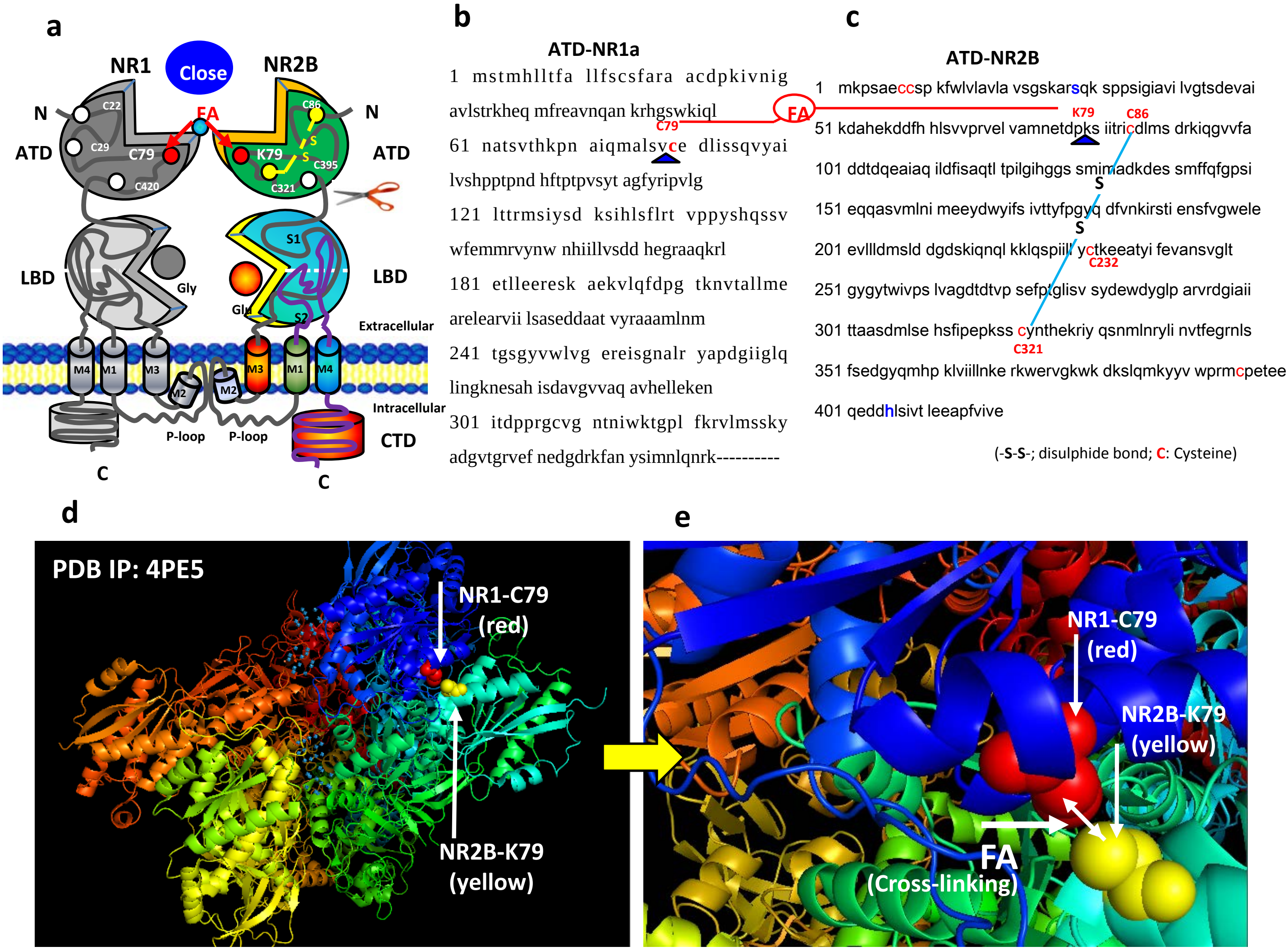

Supplementary Figure 4. Excessive FA cross-links C79 residue of NR1 and K79 site of NR2B to block NMDA-receptor. (**a**) The model of excessive FA-blocking the complex of NR1 and NR2B. (**b, c**) The sequences of amino-terminal domain (ATD) of rat NR1 and NR2B. (**d**) The 3D crystal structure of rat NR1/NR2B (PDB IP: 4PE5). (**E**) The model of excessive FA cross-linking C79 residue of NR1 and K79 site of NR2B.

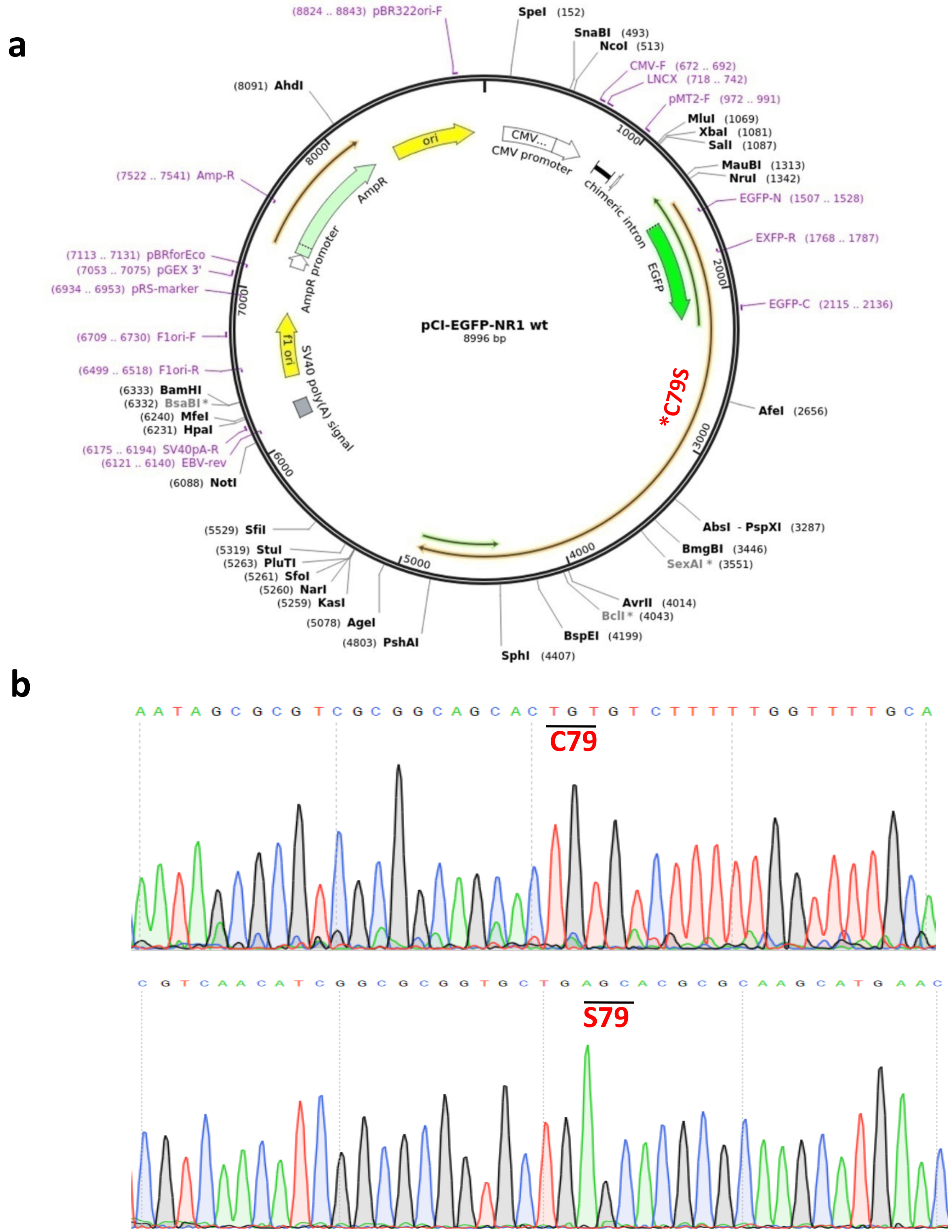

Supplementary Figure 5. The identification of mutation in C79S site of NR1 of wild-type rat. (a) The plasmid of pci-EGFP-NR2B (8996 bp). (**b)** The DNA sequence of mutation in C79S (Cys codon: TGT; Ser codon: AGC) site of NR1 examined by gene sequencing.

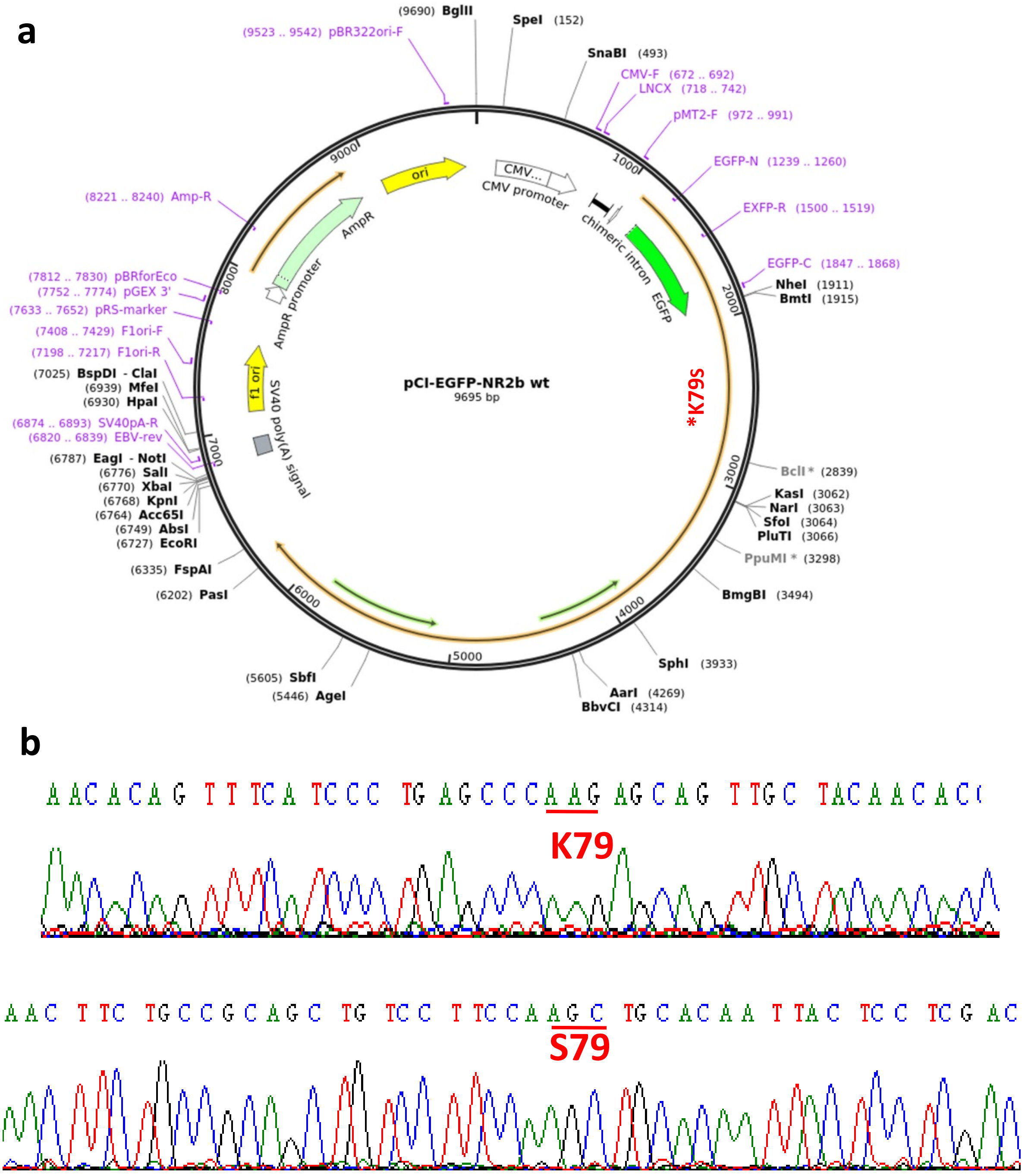

Supplementary Figure 6. The identification of mutation in K79S site of NR2B of wild-type rat. (a) The plasmid of pci-EGFP-NR2B (9695 bp). **(b)** The DNA sequence of mutation in K79S (Lys codon: AAG; Ser codon: AGC) site of NR2B examined by gene sequencing.

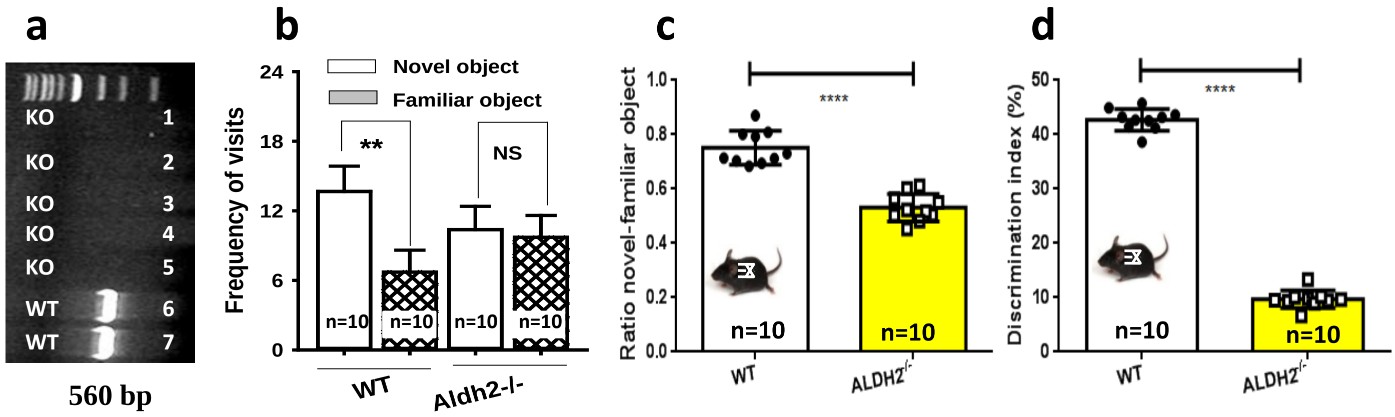

Supplementary Figure 7. *Aldh2*$^{-/-}$ mice exhibit a marked decline in the novel object recognition task performance than wild-type mice. (**a**) *Aldh2* gene in the mice was identified by PCR. (**b**) Frequency of visits to the novel and familiar objects for wild-type mice (WT) and ***Aldh2***$^{-/-}$ mice. n = 10. (**c**) Ratio of time spent with the novel object in relation to the familiar object. (**d**) Discrimination index. The data are expressed as the mean ± standard deviation (s.e.m.). $^{**}p < 0.01$. NS: no statistical significance.

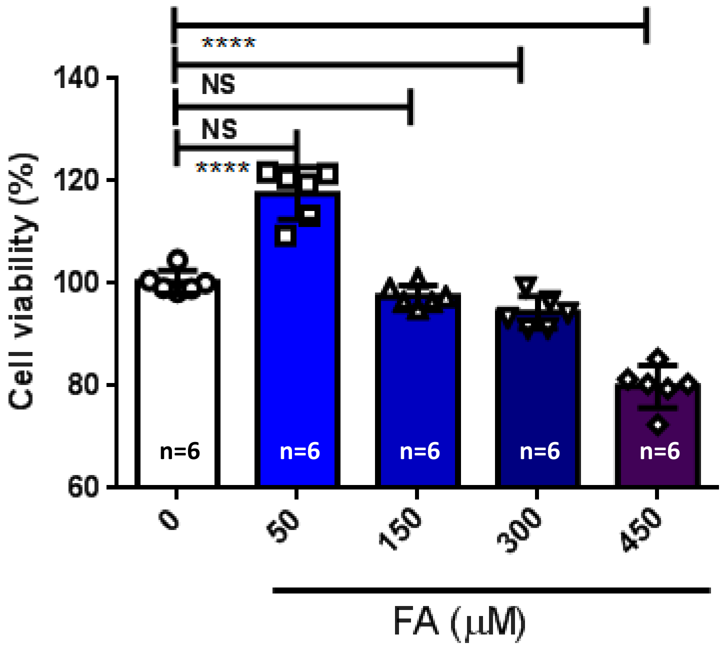

Supplementary Figure 8. The effects of FA at different concentrations on cell viability in the cultured human SY5Y cells quantified by the Cell Counting Kit 8 (CCK-8). *n* = 6 cultures per treatment. The data are expressed as the mean ± standard deviation (s.e.m.). $^{**}p < 0.01$. NS: no statistical significance.

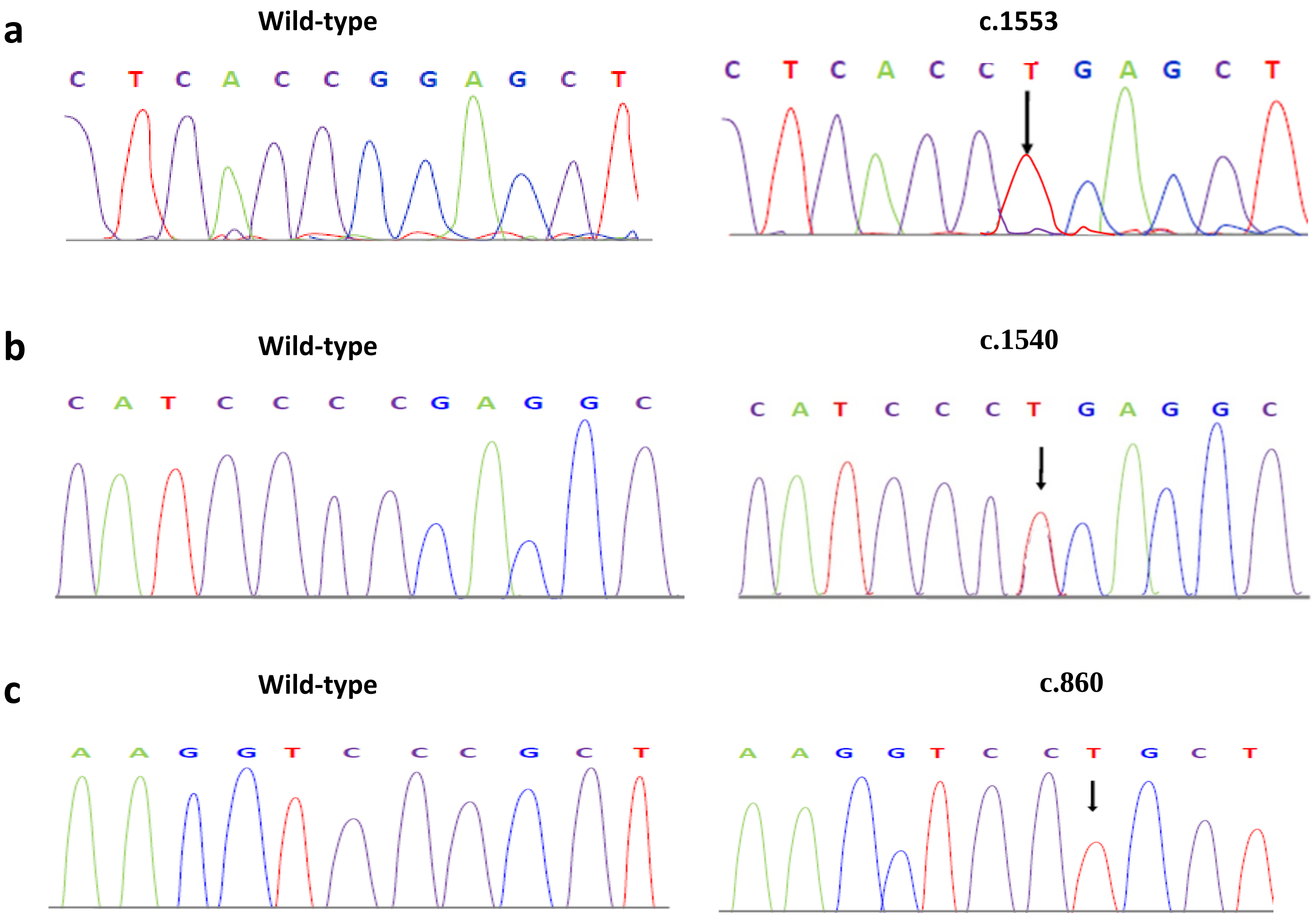

Supplementary Figure 9. Sarcosinemia children (a rare neurodegenerative disease) with *SARDH* mutations at c.1553, c.1540, and c.860, respectively. The arrows indicate the site of the mutation.

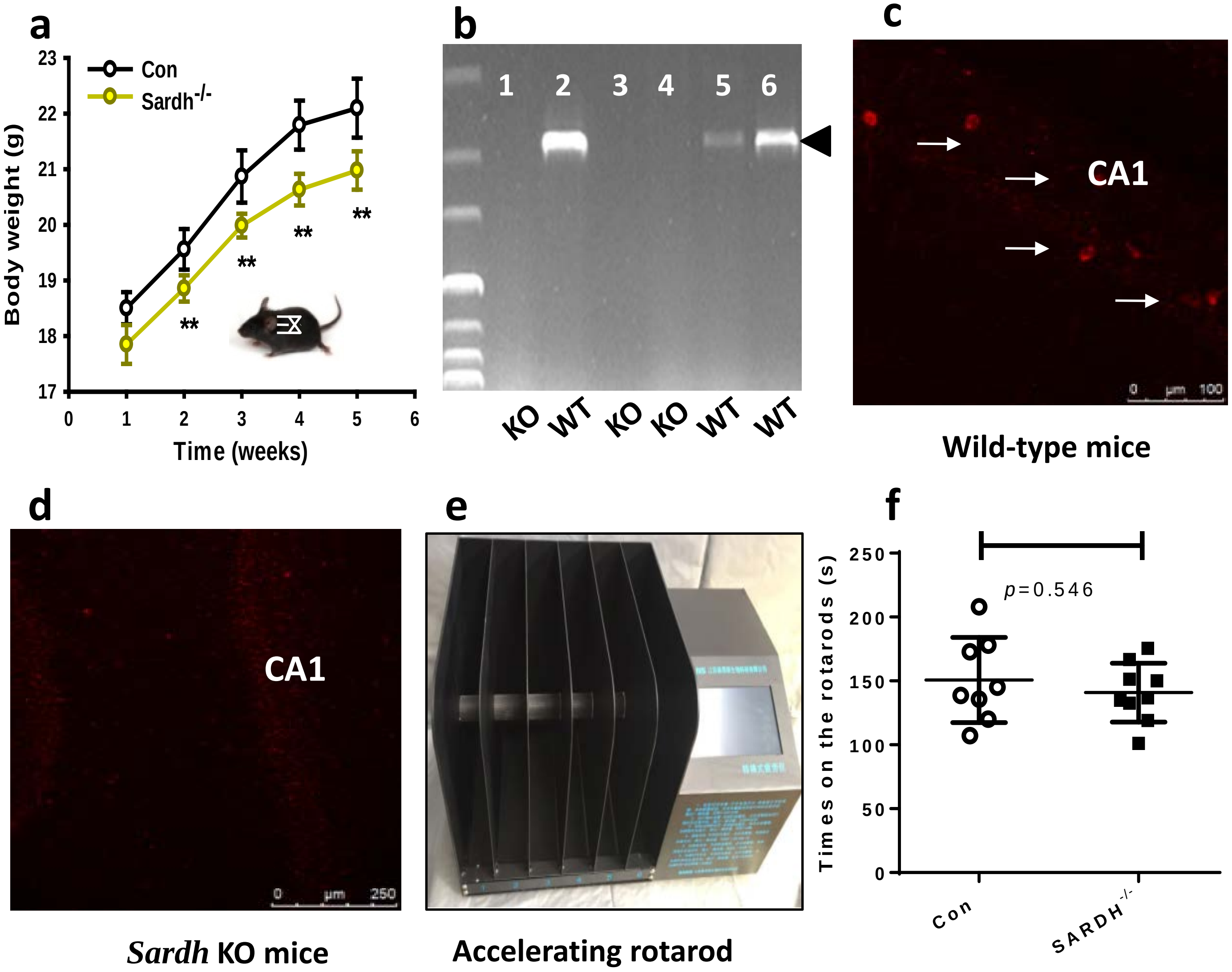

Supplementary Figure 10. Knockout of *Sardh* affects body weight and SARDH expression in mice. (**a**) Changes in body weight of ***Sardh*** KO mice and control mice from week 1 to 5. (**b**) *Sardh* gene in the mice was identified by PCR. (**c, d**) Low expression of SARDH proteins in the hippocampus of ***Sardh*** $^{-/-}$ mice than wild-type mice. (**e, f**) The motor abilities of ***Sardh*** $^{-/-}$ mice (n=9) than wild-type mice (n=8) examined by using accelerating rotarod (4 to 70 rpm, 5 minutes). Data are expressed as the mean± standard error (s.e.m.). ***p* < 0.01.

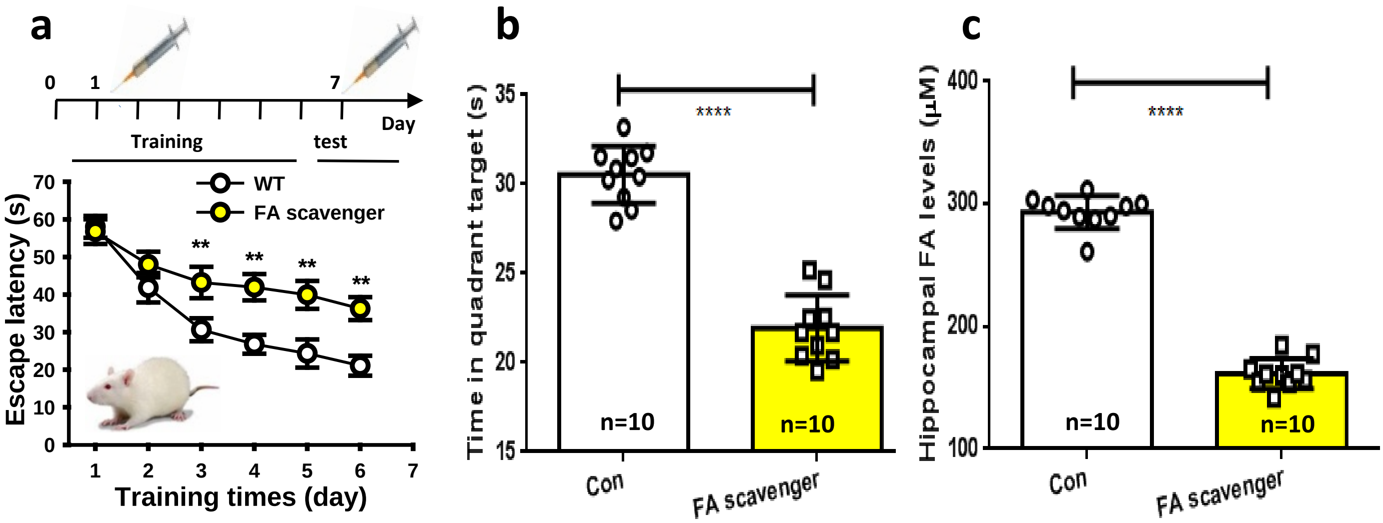

Supplementary Figure 11. Formaldehyde deficiency by injecting of FA scavenger induces spatial memory deficits in healthy wild-type SD rats. (**a**) Through the MWM test, *Post hoc* analyses of the mean escape latency values for the rats injected with FA scavenger ($NaHSO_3$, 300 μM) were significant longer than control groups on day 3 ($F_{(1, 18)} = 2.18$, $p = 0.005$), day 4 ($F_{(1, 18)} = 4.22$, $p = 0.001$), day 5 ($F_{(1, 18)} = 5.87$, $p = 0.003$) and day 6 ($F_{(1, 18)} = 5.19$, $p = 0.004$). (**b**) Rats injected with FA scavenger had shorter time of staying in target quadrant. n = 10. (**c**) Hippocampal FA levels detected by Fluo-HPLC, n = 10. Data are expressed as the mean ± standard error (s.e.m.). **$p < 0.01$.

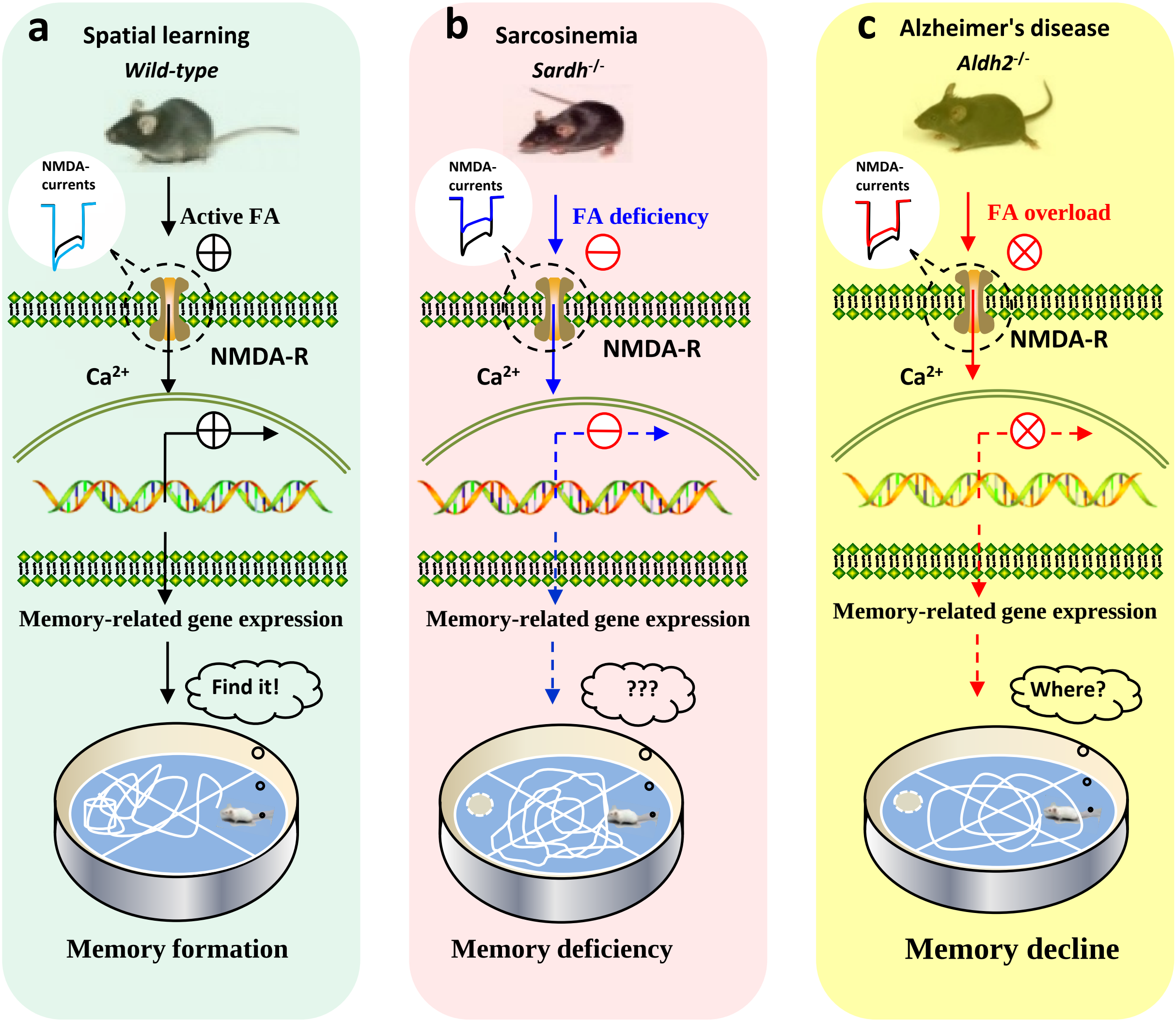

Supplementary Figure 12. Endogenous FA controls memory formation by regulating NMDA-R dually. Briefly, spatial learning elicited a rapid generation of active FA in rat hippocampus; the concentrations attained were sufficient to facilitate NMDA-currents and enhanced memory formation. However, brain FA deficiency in sarcosinemia children associated with *SARDH* mutation or in *Sardh*$^{-/-}$ mice also led to cognitive deficits by reducing NMDA-currents. In addition, excess FA impaired memory in *aldh2*$^{-/-}$ mice and AD patients with *ALDH2* mutation by suppressing NMDA-R.

Supplementary Table 1. Severe FA overload and cognitive decline in AD patients compared with healthy controls.

Test items	Control (n = 87)	AD (n= 71)	P value
Age (Years old)	71.69±2.75	73.52±3.63	> 0.05
Sex (Man/Female)	25/25	23/22	> 0.05
Education (Years)	7.32±2.78	7.09±2.07	> 0.05
MMSE scores	27.68±1.65	14.26±2.09	< 0.01
Blood SA (μM)	22.14±2.34	21.67±1.78	> 0.05
Urine SA (μM)	14.84±1.97	13.46±2.46	> 0.05
Blood FA (μM)	75.45±3.58	88.63±3.45	< 0.01
Urine FA (μM)	23.32±4.38	45.37±5.38	< 0.01
ALDH2 activity (mOD/min)	2.36±0.08	0.48±0.05	< 0.01

SA: sarcosine; FA: formaldehyde; AD: Alzheimer's disease; MMSE: Mini-Mental State Examination

Supplementary Table 2. Hardy-Weinberg equilibrium identified genotyping of *ALDH2* in AD patients and age-matched healthy controls.

Groups	N	Genotypes			alleles	
		GG	GA	AA	G	A
AD (n, %)	71	16 (22.53)	20 (28.16)	35 (49.29)	52 (36.61)	90 (63.38)
Con (n, %)	87	51 (58.62)	23 (26.43)	13 (14.94)	125 (71.83)	49 (28.16)
χ^2	-	6.09	6.61	-	39.36	-
P value	-	0.014	0.01	-	< 0.0001	-
OR (95% CI)	-	0.36 (0.15-0.82)	0.32 (0.13-0.77)	-	4.41 (2.74-7.10)	-

OR: odd ratio; CI: confidential interval; “-”: No data; AD: Alzheimer's disease

Supplementary Table 3. Severe FA deficiency and cognitive impairments in sarcosinemia children compared with healthy controls.

Test items	Control (n =31)	Sarcosinemia (n = 11)	P value
Age (Years old)	6.08±1.36	6.28±2.03	> 0.05
Sex (Man/Female)	16/15	6/5	> 0.05
Weight (Kg)	20.79±1.32	17.25±1.15	< 0.01
WISC scores	103.35±14.57	66.48±9.69	< 0.01
Blood SA (μM)	21.05±2.61	83.68±3.55	< 0.01
Urine SA (μM)	13.47±1.38	55.49±2.52	< 0.01
Blood FA (μM)	82.05±4.34	58.49±2.81	< 0.01
Urine FA (μM)	30.26±2.65	11.64±1.79	< 0.01
SARDH activity (μM/min)	5.32±0.64	1.38±0.07	< 0.01

WISC: Wechsler Intelligence Scale for Children; SA: sarcosine; FA: formaldehyde
